# Supplementary material for: Combination of antiviral drugs inhibits SARS-CoV-2 polymerase and exonuclease and demonstrates COVID-19 therapeutic potential in viral cell culture
Source: Commun Biol. 2022 Feb 22;5:154. doi: 10.1038/s42003-022-03101-9 (PMC8863796; doi:10.1038/s42003-022-03101-9)
Supplement: Supplementary file 1 — Supplementary Information [file 42003_2022_3101_MOESM1_ESM.pdf]

SUPPLEMENTARY INFORMATION

Combination of Antiviral Drugs to Inhibit SARS-CoV-2 Polymerase and Exonuclease Demonstrates COVID-19 Therapeutic Potential

Xuanting Wang[1,2,#], Carolina Q. Sacramento[3,4,#], Steffen Jockusch[1,5,#], Otávio Augusto Chaves[3,4], Chuanjuan Tao[1,2], Natalia Fintelman-Rodrigues[3,4], Minchen Chien[1,2], Jairo R. Temerozo[6,7], Xiaoxu Li[1,2], Shiv Kumar[1,2], Wei Xie[8], Dinshaw J. Patel[8], Cindy Meyer[9], Aitor Garzia[9], Thomas Tuschl[9], Patrícia T. Bozza[3], James J. Russo[1,2], Thiago Moreno L. Souza[3,4,†,*], Jingyue Ju[1,2,10,†,*]

1 - Center for Genome Technology and Biomolecular Engineering, Columbia University, New York, NY 10027, USA.

2 - Department of Chemical Engineering, Columbia University, New York, NY 10027, USA.

3 - Laboratory of Immunopharmacology, Oswaldo Cruz Institute (IOC), Oswaldo Cruz Foundation (Fiocruz), Rio de Janeiro, RJ, Brazil.

4 - National Institute for Science and Technology for Innovation on Diseases of Neglected Population (INCT/IDN), Center for Technological Development in Health (CDTS), Oswaldo Cruz Foundation (Fiocruz), Rio de Janeiro, RJ, Brazil.

5 - Department of Chemistry, Columbia University, New York, NY 10027, USA.

6 - Laboratory on Thymus Research, Oswaldo Cruz Institute (IOC), Oswaldo Cruz Foundation (Fiocruz), Rio de Janeiro, RJ, Brazil.

7 - National Institute for Science and Technology on Neuroimmunomodulation (INCT/NIM), Oswaldo Cruz Institute (IOC), Oswaldo Cruz Foundation (Fiocruz), Rio de Janeiro, RJ, Brazil.

8 - Laboratory of Structural Biology, Memorial Sloan-Kettering Cancer Center, New York, NY 10065, USA.

9 - Laboratory of RNA Molecular Biology, Rockefeller University, New York, NY 10065.

10 - Department of Molecular Pharmacology and Therapeutics, Columbia University, New York, NY 10032, USA.

# - These authors contributed equally

† - These authors jointly supervised this work

*To whom correspondence should be addressed. Email: dj222@columbia.edu (JJ); tmoreno@cdts.fiocruz.br (TMLS)

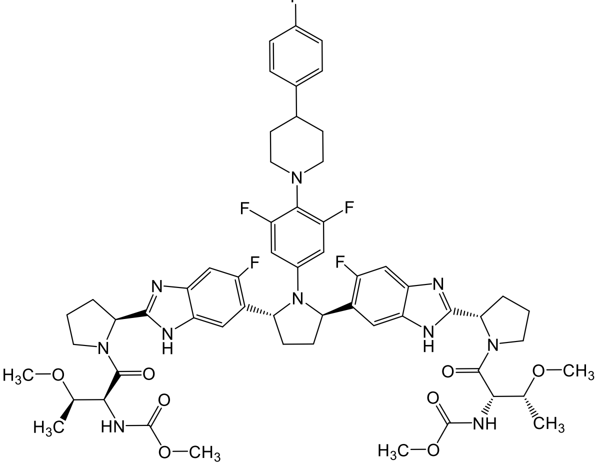

Pibrentasvir

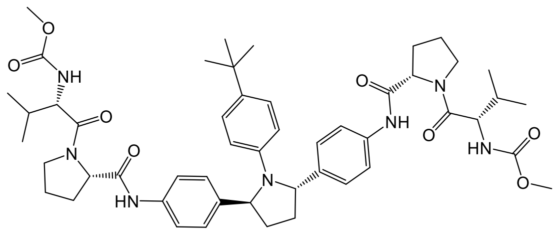

Ombitasvir

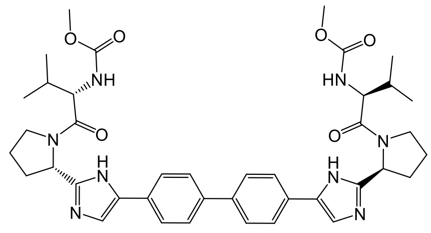

Daclatasvir

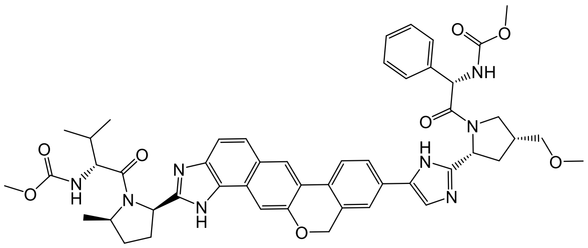

Velpatasvir

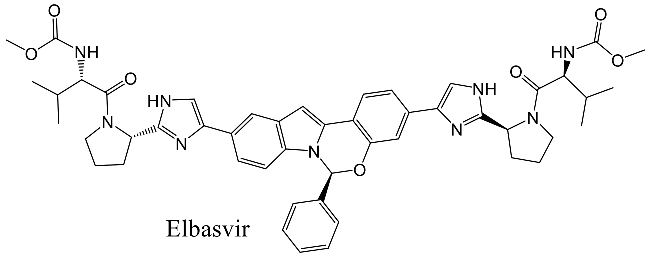

Elbasvir

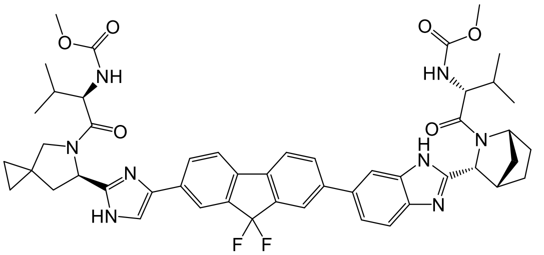

Ledipasvir

Fig. S-1 Structures of HCV NS5A inhibitors: Pibrentasvir, Ombitasvir, Daclatasvir, Velpatasvir, Elbasvir and Ledipasvir.

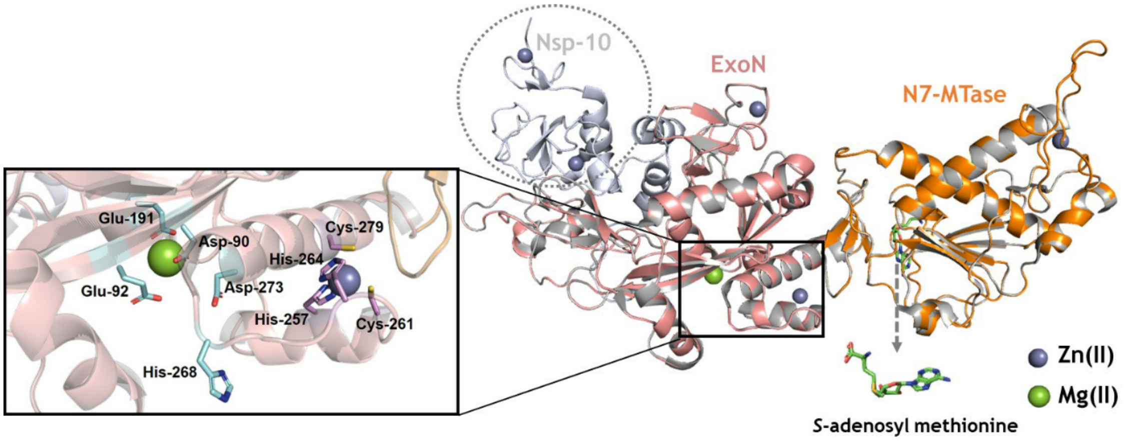

Fig. S-2 Superposition of the SARS-CoV nsp14 (PDB code: 5C8T, in gray) and SARS-CoV-2 nsp14 model (beige and orange for ExoN and N7-MTase domains, respectively). Selected amino acid residues in the exonuclease catalytic site are represented as stick representations in cyan. The co-substrate involved in methyltransferase, *S*-adenosyl methionine (SAM), is represented using a stick model in green. The hydrogen atoms were omitted for better clarity. The oxygen (red), nitrogen (dark blue) and sulfur atoms (yellow) are presented in the stick structure. The Mg^{++} (green) and Zn^{++} (indigo blue) ions are represented as spheres.

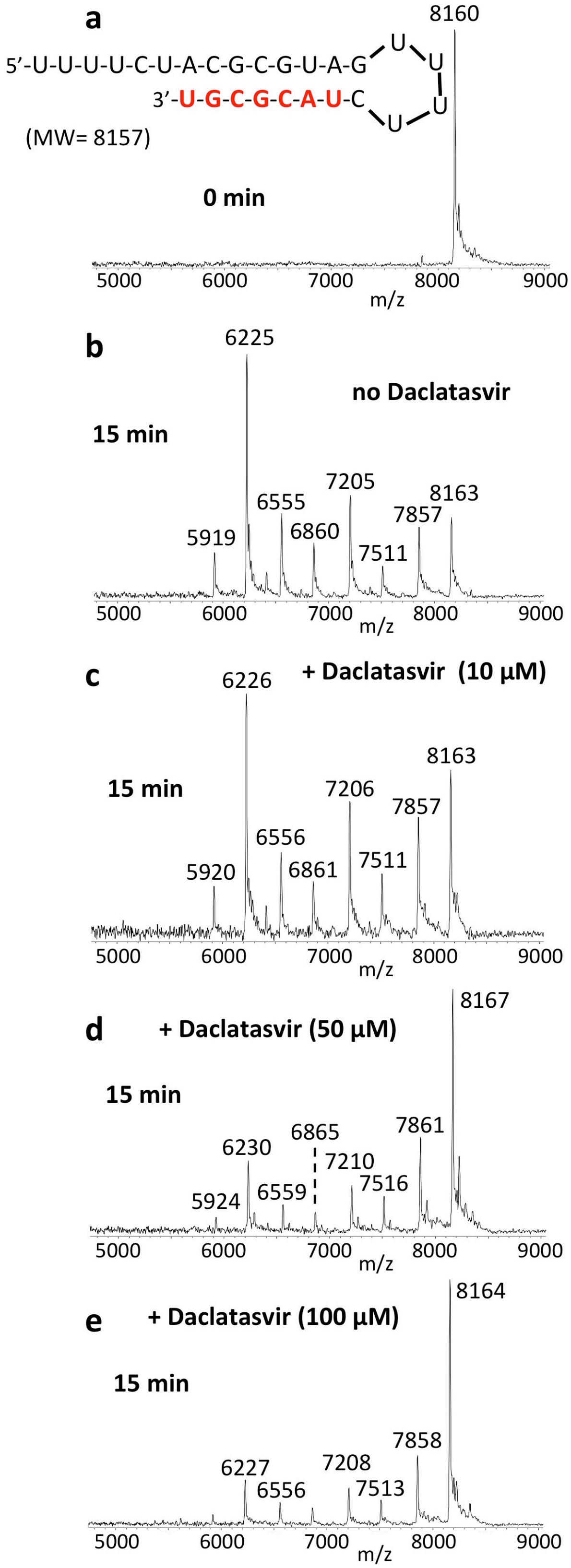

Fig. S-3 Inhibition of SARS-CoV-2 exonuclease activity by Daclatasvir. A mixture of 500 nM RNA (sequence shown at the top of the figure) and 50 nM SARS-CoV-2 pre-assembled exonuclease complex (nsp14/nsp10) was incubated in buffer solution at 37 °C for 15 min in the absence (b) and presence of varying amounts of Daclatasvir dihydrochloride (c-e). In this set of experiments, the water-soluble dihydrochloride form of Daclatasvir was used without DMSO. The RNA (a) and the products of the exonuclease reaction (b-e) were analyzed by MALDI-TOF MS. The signal intensity was normalized to the highest peak. The peak at 8160 Da corresponds to the intact RNA (8157 Da expected). In the absence of Daclatasvir, exonuclease activity caused nucleotide cleavage from the 3'-end of the RNA as shown by the 7 lower molecular weight fragments corresponding to cleavage of 1-7 nucleotides (b). With increasing amounts of Daclatasvir, exonuclease activity was reduced as shown by the reduced intensities of the fragmentation peaks and increased intact RNA peak (c-e).

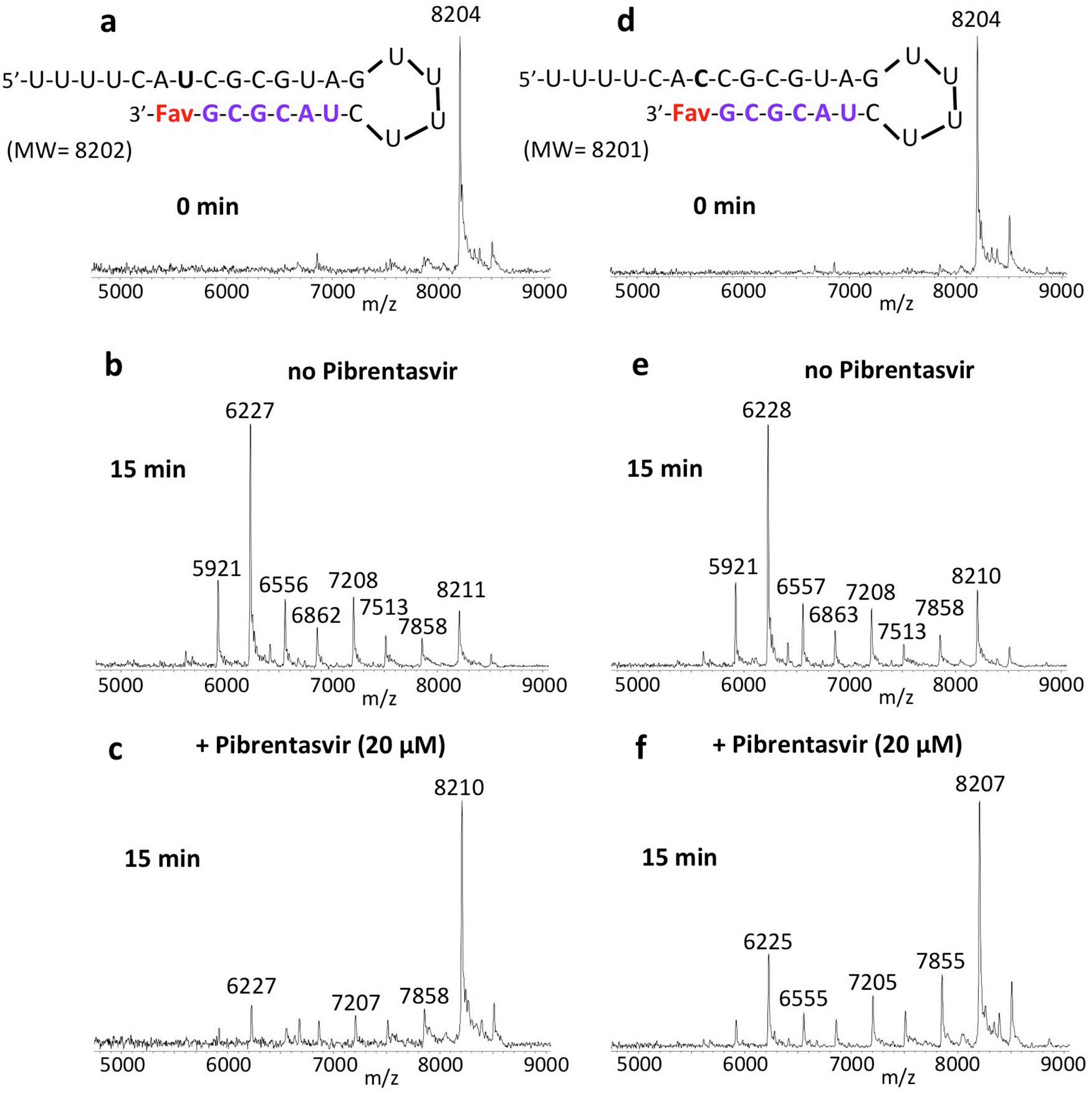

Fig. S-4 Inhibition of SARS-CoV-2 exonuclease activity by Pibrentasvir for Favipiravir (Fav) terminated RNAs. A mixture of 500 nM RNAs (sequences shown at the top of the figure) and 50 nM SARS-CoV-2 pre-assembled exonuclease complex (nsp14/nsp10) were incubated in buffer solution at 37 °C for 15 min in the absence (b, e) and presence of 20 µM Pibrentasvir (c, f). The intact RNAs (a, d) and the products of the exonuclease reactions (b, c, e, f) were analyzed by MALDI-TOF MS. The signal intensity was normalized to the highest peak. In the absence of Pibrentasvir, exonuclease activity caused nucleotide cleavage from the 3'-end of the RNA as shown by the lower molecular weight fragments corresponding to cleavage of 1-7 nucleotides (b, e). When 20 µM Pibrentasvir was added, exonuclease activity was reduced as shown by the reduced intensities of the fragmentation peaks and increased peak height of the intact RNA (c, f). These results indicate that the SARS-CoV-2 exonuclease activity is substantially inhibited by Pibrentasvir for Favipiravir terminated RNAs.

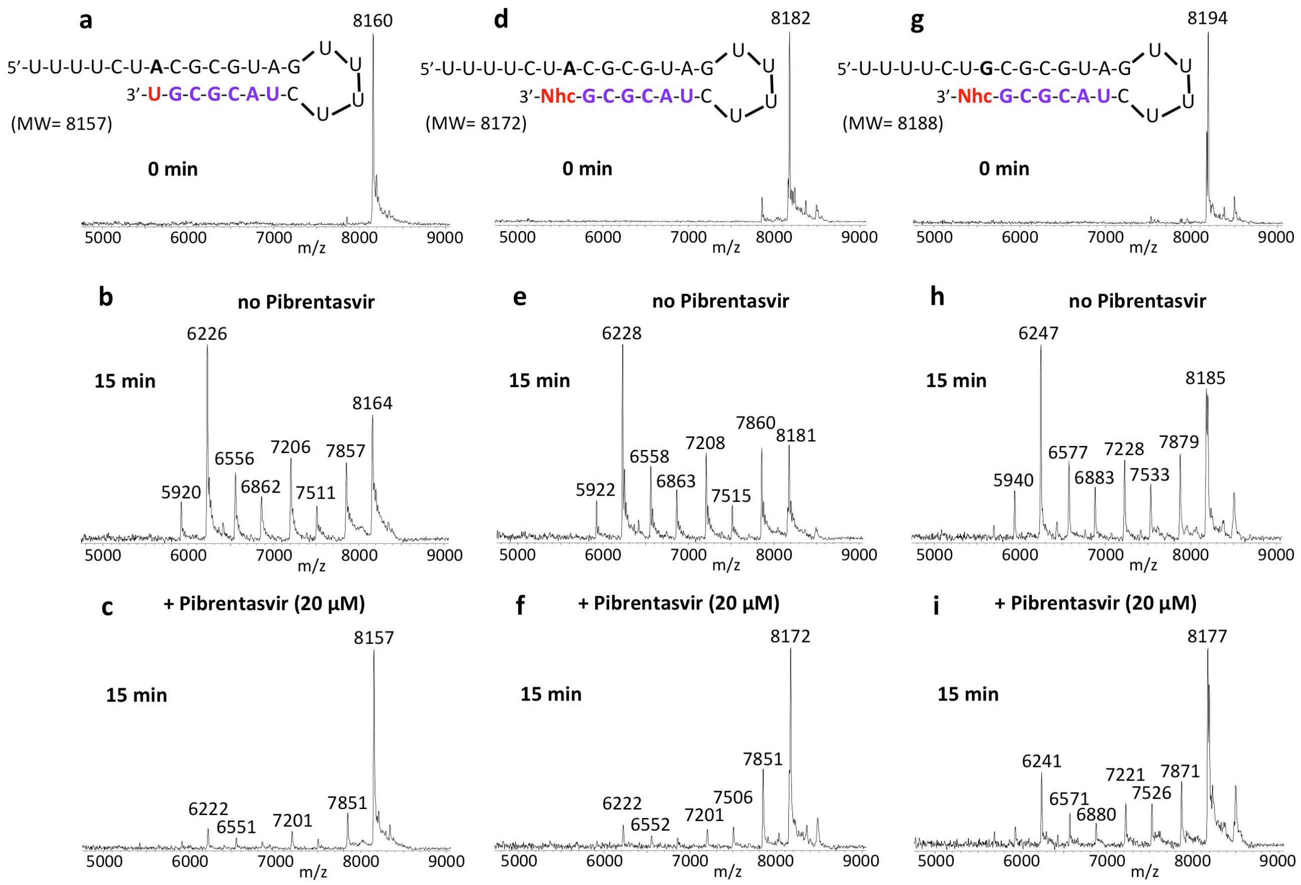

Fig. S-5 Inhibition of SARS-CoV-2 exonuclease activity by Pibrentasvir for natural RNA and N⁴-hydroxycytidine (Nhc) terminated RNAs. A mixture of 500 nM RNAs (sequences shown at the top of the figure) and 50 nM SARS-CoV-2 pre-assembled exonuclease complex (nsp14/nsp10) were incubated in buffer solution at 37 °C for 15 min in the absence (b, e, h) and presence of 20 µM Pibrentasvir (c, f, i). The intact RNAs (a, d, g) and the products of the exonuclease reactions (b, c, e, f, h, i) were analyzed by MALDI-TOF MS. The signal intensity was normalized to the highest peak. In the absence of Pibrentasvir, exonuclease activity caused nucleotide cleavage from the 3'-end of the RNA as shown by the lower molecular weight fragments corresponding to cleavage of 1-7 nucleotides (b, e, h). When 20 µM Pibrentasvir was added, exonuclease activity was reduced as shown by the reduced intensities of the fragmentation peaks and increased peak height of the intact RNA (c, f, i). These results indicate that the SARS-CoV-2 exonuclease activity is substantially inhibited by Pibrentasvir for NHC terminated RNAs.

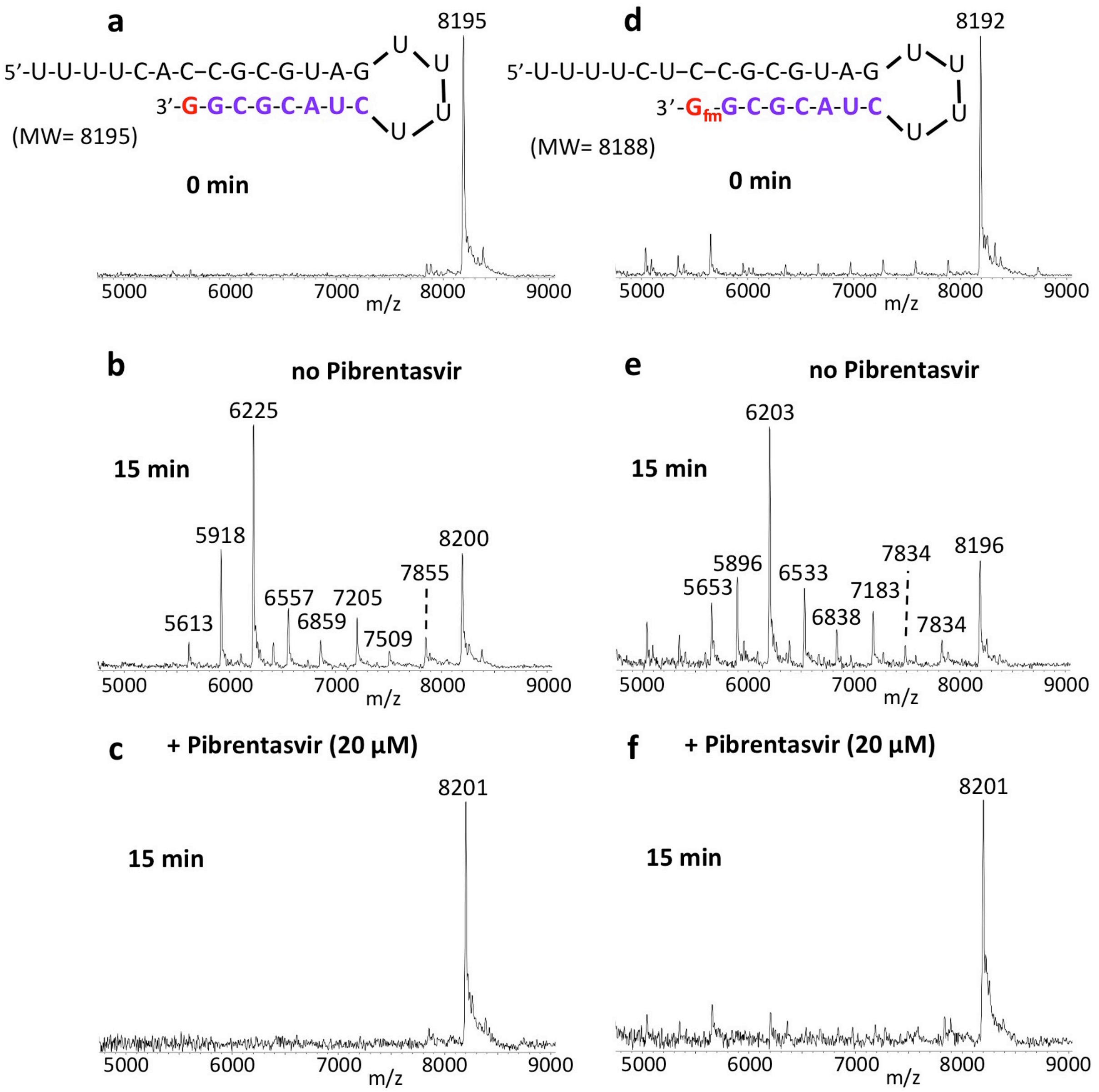

Fig. S-6 Inhibition of SARS-CoV-2 exonuclease activity by Pibrentasvir for natural RNA and AT-9010 (G_{fm}) terminated RNA. A mixture of 500 nM RNAs (sequences shown at the top of the figure) and 50 nM SARS-CoV-2 pre-assembled exonuclease complex (nsp14/nsp10) were incubated in buffer solution at 37 °C for 15 min in the absence (b, natural RNA; e, G_{fm} terminated RNA) and presence of 20 µM Pibrentasvir (c, natural RNA; f, G_{fm} terminated RNA). The intact RNAs (a, d) and the products of the exonuclease reactions (b-f) were analyzed by MALDI-TOF MS. The signal intensity was normalized to the highest peak. In the absence of Pibrentasvir, exonuclease activity caused nucleotide cleavage from the 3'-end of the natural RNA as shown by the lower molecular weight fragments corresponding to cleavage of 1-8 nucleotides (b, e). When 20 µM Pibrentasvir was added, exonuclease activity was almost completely abolished as shown by the near absence of the fragmentation peaks and increased peak height of the intact RNA (c, f). These results indicate that the SARS-CoV-2 exonuclease activity is substantially inhibited by Pibrentasvir for both natural and AT-9010 (G_{fm}) terminated RNA.

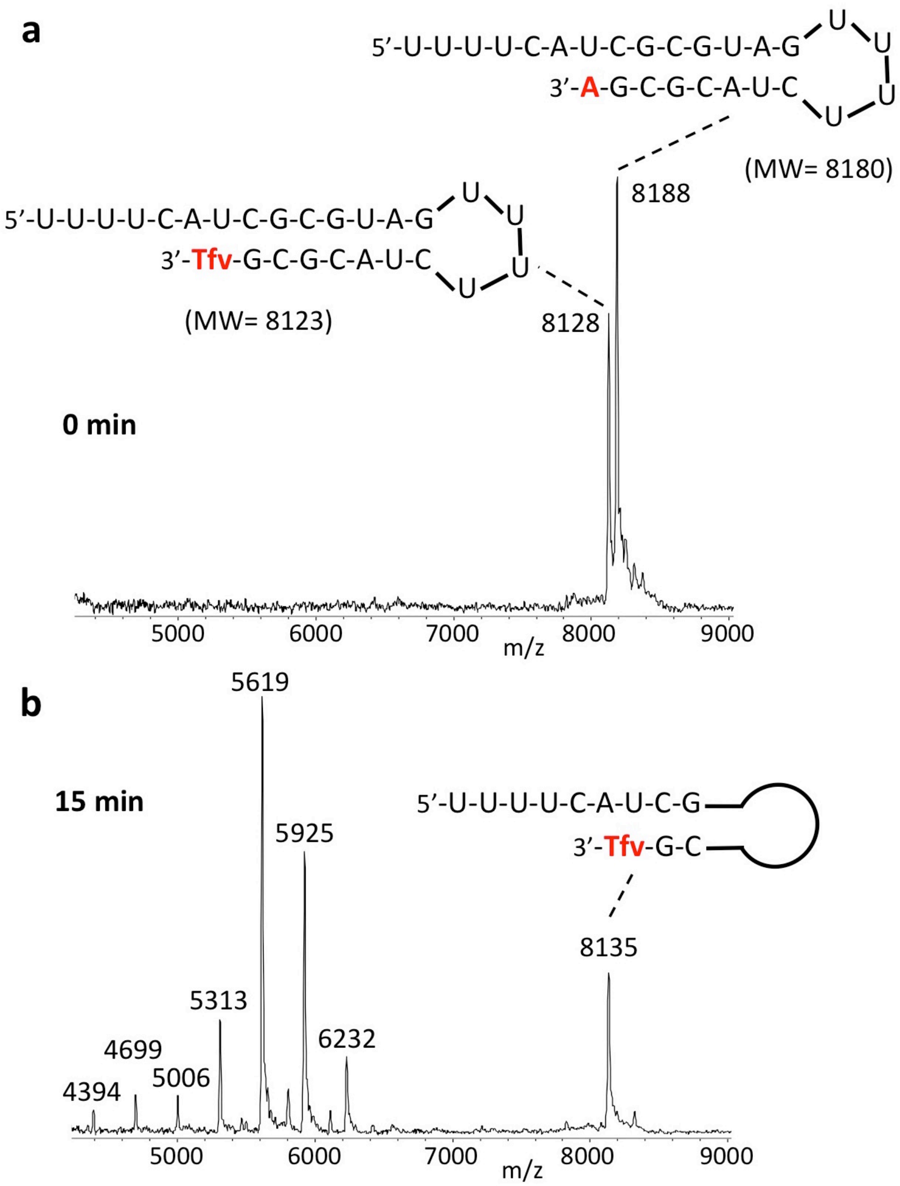

Fig. S-7 SARS-CoV-2 exonuclease activity for a mixture of natural RNA and Tenofovir (Tfv) terminated RNA. A mixture of ~500 nM RNAs (sequences shown at the top of the figure) and 50 nM SARS-CoV-2 pre-assembled exonuclease complex (nsp14/nsp10) were incubated in buffer solution at 37 °C for 15 min (b). The untreated RNA mixture (0 min) (a) and the products of the exonuclease reactions (b) were analyzed by MALDI-TOF MS. Exonuclease activity caused nucleotide cleavage from the 3'-end of the natural RNA as shown by the lower molecular weight fragments corresponding to cleavage of up to 12 nucleotides (b). No detectable MS peak was observed for the full-length natural RNA. However, a large MS peak for intact Tfv terminated RNA was observed at 8135 Da (b) demonstrating that Tfv terminated RNA shows high resistance to SARS-CoV-2 exonuclease activity compared to natural RNA.

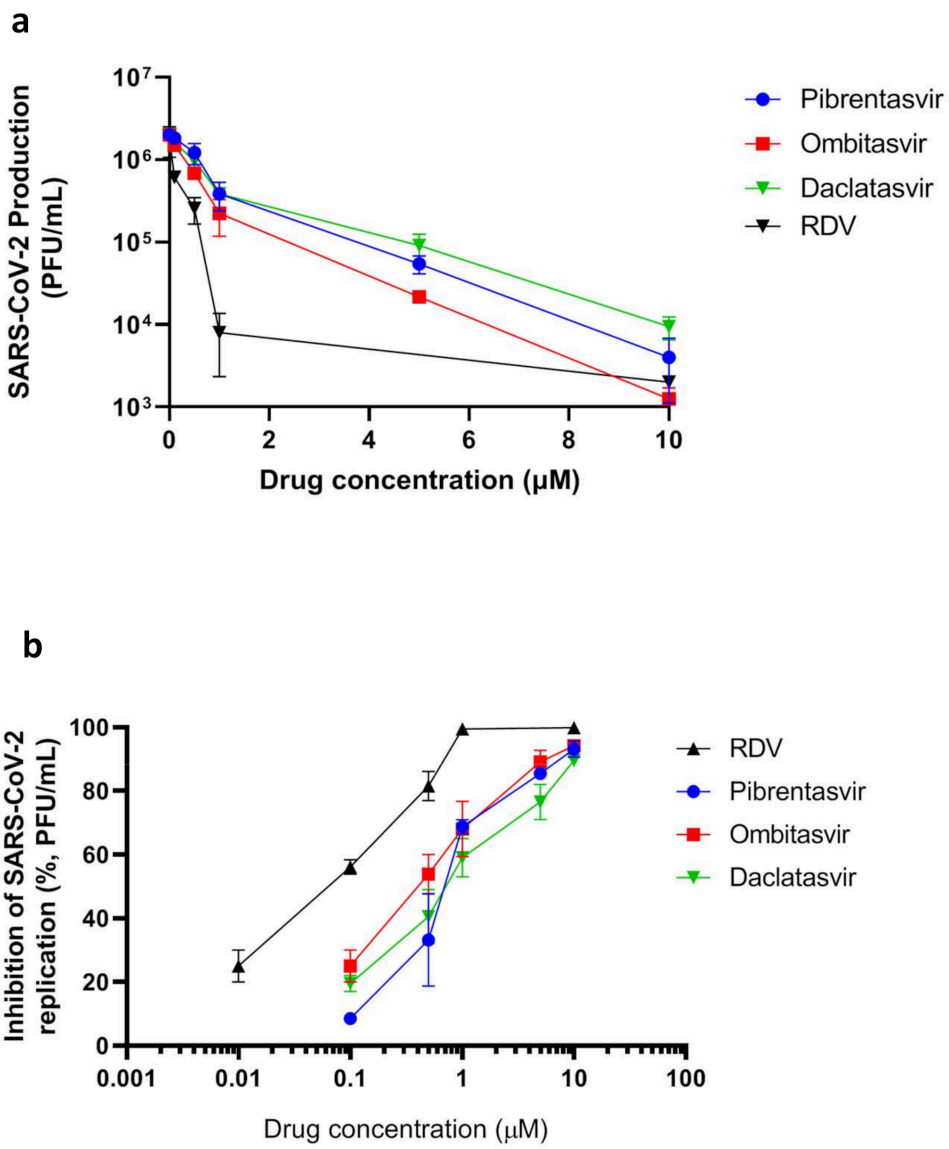

Fig. S-8 Antiviral activity of Pibrentasvir, Ombitasvir and Daclatasvir against SARS-CoV-2. Calu-3 cells, at a density of 5×10^5 cells/well in 48-well plates, were infected with SARS-CoV-2 at a MOI of 0.1, for 1 h at 37 °C. An inoculum was removed and cells were washed and incubated with fresh DMEM containing 2% FBS and the indicated concentration of the drugs, including Remdesivir (RDV) as the control. Supernatants were assessed after 48-72 h. Viral replication in the culture supernatant was measured as PFU/mL by titering in Vero E6 cells. Results are displayed as virus titers (a) and percentage of inhibition (b). The data represent means ± SEM of three independent experiments.

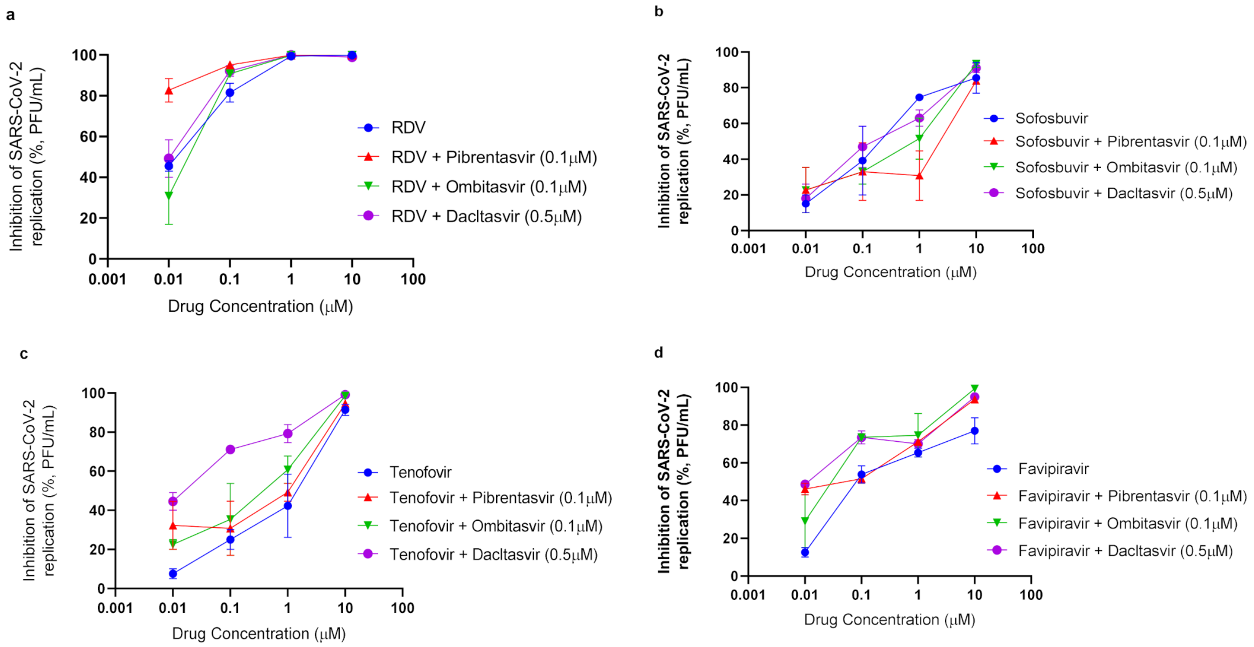

Fig. S-9 Antiviral activity of combinations of SARS-CoV-2 polymerase and exonuclease inhibitors. Calu-3 cells, at a density of 5×10^5 cells/well in 48-well plates, were infected with SARS-CoV-2 at a MOI of 0.1, for 1 h at 37 °C. An inoculum was removed and cells were washed and incubated with fresh DMEM containing 2% FBS and the indicated concentration of Remdesivir (RDV) (a), Sofosbuvir (b), Tenofovir (c), and Favipiravir (d), alone and in combination with the HCV NS5A inhibitors. Supernatants were assessed after 48-72 h. Viral replication in the culture supernatant was measured as PFU/mL by titering in VeroE6 cells. Results are displayed as inhibition of virus replication over untreated control. The data represent means ± SEM of three independent experiments.

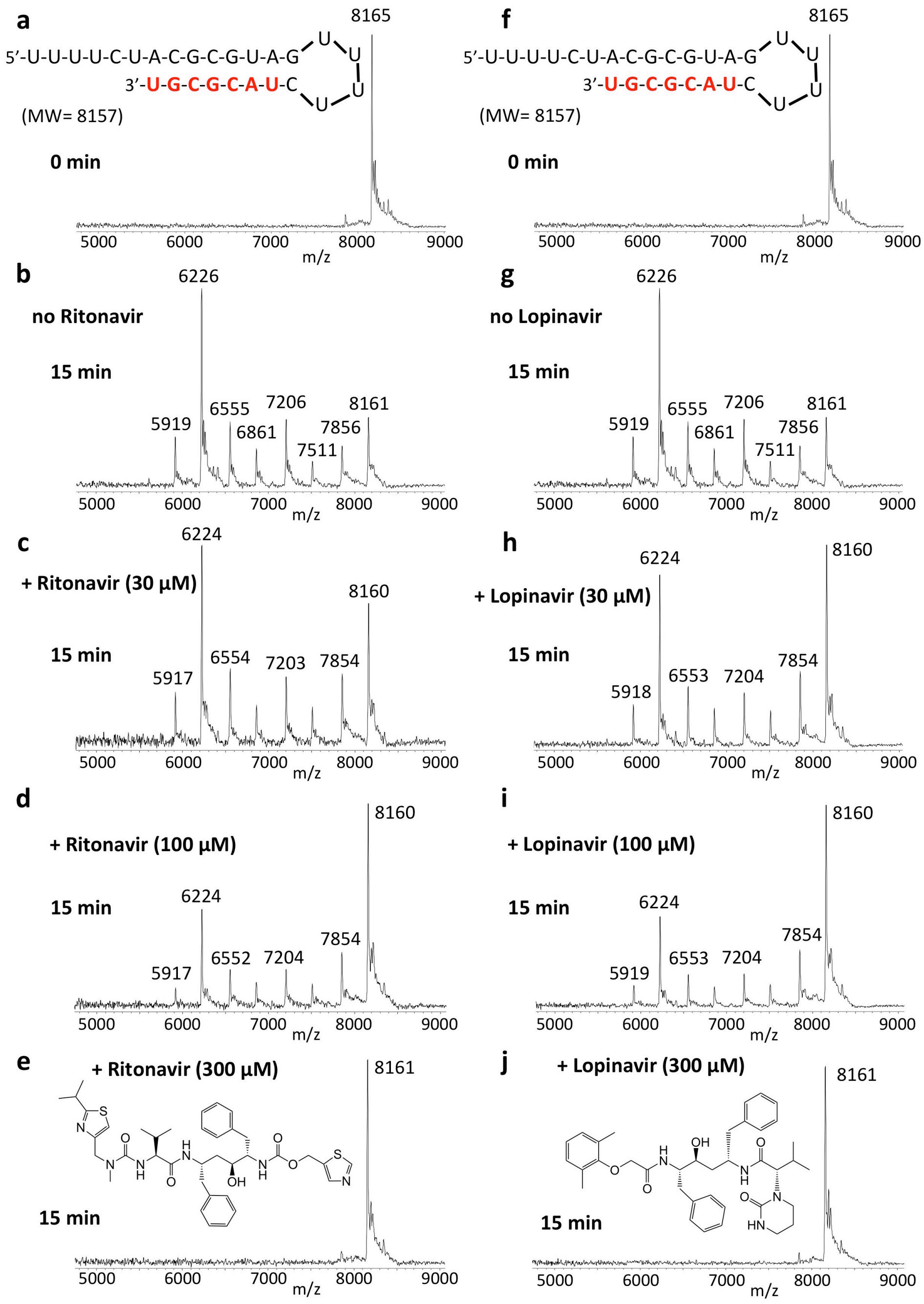

Fig. S-10 Inhibition of SARS-CoV-2 exonuclease activity by Ritonavir and Lopinavir. A mixture of 500 nM RNA (sequence shown at the top of the figure) and 30 nM SARS-CoV-2 pre-assembled exonuclease complex (nsp14/nsp10) was incubated in buffer solution at 37 °C for 15 min in the absence (b, g) and presence of 30 µM (c, h), 100 µM (d, i) or 300 µM (e, j) Ritonavir or Lopinavir (structures shown at the bottom of the figure). The RNA (a, f) and the products of the exonuclease reactions (b-e, g-j) were analyzed by MALDI-TOF MS. The signal intensity was normalized to the highest peak. The peak at 8165 Da corresponds to the intact RNA (8157 Da expected). In the absence of the NS5A inhibitors, exonuclease activity caused nucleotide cleavage from the 3'-end of the RNA as shown by the 7 lower molecular weight fragments corresponding to cleavage of 1-7 nucleotides (b, g). In the presence of Ritonavir and Lopinavir, exonuclease activity was inhibited as shown by the reduced intensities of fragmentation peaks and increased intensity of the intact RNA peak (c-e, h-j).

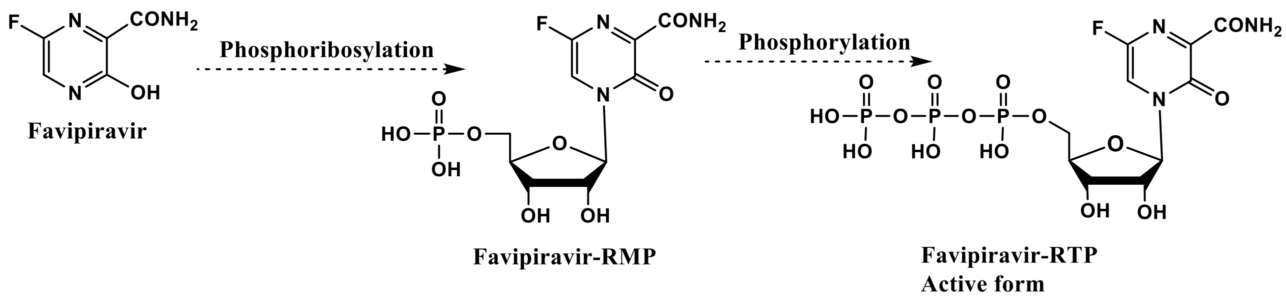

Fig. S-11 Mechanism of activation of Favipiravir. The nucleotide Favipiravir undergoes a phosphoribosylation reaction to form Favipiravir-ribofuranosyl-5'-monophosphate (RMP) followed by further phosphorylation to form the active triphosphate Favipiravir-ribofuranosyl-5'-triphosphate (RTP).

Table S-1 *In vitro* pharmacological parameters for representative viral RNA polymerase and HCV NS5A inhibitors against SARS-CoV-2 production in Calu-3 cells.

	**EC_{50}**	**CC_{50}**	**SI**
**Drug**	**[μM]**	**[μM]**	
Remdesivir	0.09 ± 0.002	480 ± 55	5333
Sofosbuvir	6.2 ± 0.3	512 ± 34	82
Tenofovir (TDF)	4.3 ± 2.1	91 ± 25	21
Favipiravir	7.8 ± 1.2	670 ± 118	85
Pibrentasvir	0.7 ± 0.2	22 ± 3	31
Ombitasvir	0.4 ± 0.05	70 ± 12	175
Daclatasvir	0.7 ± 0.08	38 ± 5	54

SI – Selectivity index = CC_{50}/EC_{50}
