# Supplementary material for: Combination of antiviral drugs inhibits SARS-CoV-2 polymerase and exonuclease and demonstrates COVID-19 therapeutic potential in viral cell culture
Source: Commun Biol. 2022 Feb 22;5:154. doi: 10.1038/s42003-022-03101-9 (PMC8863796; doi:10.1038/s42003-022-03101-9)
Supplement: Supplementary file 2 — Description of Additional Supplementary Files [file 42003_2022_3101_MOESM2_ESM.pdf]

Description of Additional Supplementary Files

File name: Supplementary Data 1
Description: Raw data for Figure 7 and Table 1.

File name: Supplementary Data 2
Description: Raw data for Figure 8.
